# Psychometric properties of the cannabis abuse screening test (CAST) in a sample of Moroccans with cannabis use

**DOI:** 10.1186/s13722-024-00459-5

**Published:** 2024-04-03

**Authors:** Hicham El Malki, Salma Ghofrane Moutawakkil, Abdelfettah El-Ammari, Mohammed El Amine Ragala, Jaouad El Hilaly, Samir El Gnaoui, Fatima El Houari, Karima El Rhazi, Btissame Zarrouq

**Affiliations:** 1 Laboratory of Epidemiology and Research in Health Sciences, Faculty of Medicine, Pharmacy, and Dental Medicine, Sidi Mohamed Ben Abdellah University, Fez, Morocco; 2 Department of Biology-Geology, Teachers Training College (Ecole Normale Superieure), Sidi Mohamed Ben Abdellah University, Fez, Morocco; 3 R.N.E Laboratory, Multidisciplinary Faculty of Taza, Sidi Mohamed Ben Abdellah University, Fez, Morocco; 4 Laboratory of Pedagogical and Didactic Engineering of Sciences and Mathematics, Regional Center of Education and Training (CRMEF), Fez, Morocco; 5 Addictology Center, Fez, Morocco

**Keywords:** Cannabis, CAST, Addictology, Psychometrics, Validity, Morocco

## Abstract

**Background:**

The Cannabis Abuse Screening Test (CAST) is a widely used screening tool for identifying patterns of cannabis use that have negative health or social consequences for both the user and others involved. This brief screening instrument has been translated into multiple languages, and several studies examining its psychometric properties have been published. However, studies on the factorial validity and psychometric properties of a Moroccan version of the CAST are not yet available. The objective of this study is to validate the CAST, translated, and adapted to the Moroccan Arabic dialect among persons with cannabis use.

**Methods:**

A total of 370 participants from an addictology center in Fez City, were selected over two phases to form the study sample. First, in phase I, exploratory factor analysis was employed to evaluate the factor structure in the pilot sample (n_1_ = 150). Subsequently, in the second phase (Phase II), confirmatory factor analysis was utilized to confirm this structure in the validation sample (n_2_ = 220). All statistical analyses were carried out using the R program.

**Results:**

The CFA unveiled a three-factor structure that showed a good overall fit (χ2/df = 2.23, RMSEA = 0.07, SRMR = 0.02, CFI = 0.99, NFI = 0.98) and satisfactory local parameters (standardized factor loadings between 0.72 and 0.88). The model demonstrates satisfactory reliability and convergent validity, as evidenced by the acceptable values of composite reliability (CR) (0.76–0.88) and average variance extracted (AVE) (0.62–0.78), respectively. The square roots of the AVE exceeded the correlations of the factor pairs, and the heterotrait-monotrait (HTMT) ratio of the correlation values was below 0.85, indicating acceptable discriminant validity.

**Conclusion:**

The reliability, convergent validity, and discriminant validity tests all demonstrated that the Moroccan version of the CAST performed well and can be considered a valid tool for screening of problematic cannabis use.

## Background

The growing prevalence of cannabis usage – the world’s leading psychoactive substance in 2020 [1] - and the potential for developing disorders associated with its use have emerged as unavoidable issues of public health and social concern [2].

According to the United Nations Office on Drugs and Crime (UNODC), in 2020, approximately 209 million people, which accounted for more than 4% of the global population aged 15 to 64, reported using cannabis. This represents a 23% increase in persons with cannabis use compared to the data from 2010 [1]. The prevalence of cannabis use varies significantly across regions, with the highest rates found in North America, Australia, New Zealand, and Africa [3].

In Morocco, known as a major global cannabis producer, the use of cannabis in the past year was estimated to be approximately 10.47% among individuals aged 15–64 [1]. Notably, the information provided shows that the prevalence of cannabis use appears to be higher among adolescents than in the adult population. The third iteration of the Mediterranean school survey project on alcohol and other drugs (MedSPAD III), designed to collect consistent data on the use of psychoactive substances among young people aged 15 to 17 and to monitor trends within and between Mediterranean countries [4], revealed that lifetime cannabis use among 15–16-year-olds was the highest among illicit drugs, with a prevalence rate of 5.8% [5]. Similarly, a study conducted in public secondary schools in Morocco’s Centre-North region reported a 6.7% prevalence of cannabis use in the past 12 months among participating students [6].

The use of cannabis among adolescents is a matter of great concern [7] due to its association with various negative outcomes. Regular cannabis use during this developmental stage has been linked to detrimental consequences, including diminished academic performance, lower educational attainment, increased risk of addiction [8], earlier onset of psychosis [2] and cognitive decline [9].

Due to the significant health risks and potential complications linked to cannabis use, it is imperative to have validated tools for the early assessment of cannabis use. These tools are essential for identifying and addressing problematic use in a timely manner, enabling effective detection and intervention at an early age [10]. Various tools have been created for screening cannabis use, both in the general population and specific clinical groups [11]. The Cannabis Abuse Screening Test (CAST), developed and validated in 2007 [12], is a screening tool that is extensively utilized in Europe [13] to identify cannabis use patterns that have negative health or social consequences for both the user and others involved [12].

The CAST comprises six items with two scoring options: the binary CAST, where respondents answer each item with a simple yes or no, resulting in a total score ranging from 0 to 6; and the full CAST, where respondents provide a five-option response for each item, allowing for a score of 0 to 4 per item and a total CAST score ranging from 0 to 24 [14].

To date, the CAST is a commonly used tool globally to identify potential problematic cannabis use. However, its validation in the Arab and Moroccan context is currently lacking, and there is a dearth of data on the Moroccan population.

Therefore, the aim of this study is to evaluate the psychometric properties of the CAST specifically in the Moroccan population who use cannabis through its validation and adaptation in the Arabic dialect and Moroccan culture.

## Methodology

### Study design

A cross-sectional study was conducted between February 2020 and June 2022 at the addictology center of Fez city, Morocco.

### Samples/Participants

A total of 370 participants were selected over two phases to form the study sample. The sample size was estimated based on the ratio of cases to variables and the strength of the factor analysis results [15]. According to the recommended guidelines for the exploratory factor analysis (EFA) a ratio of participants (N) to variables (p) set at 5:1 is used, provided that the sample size exceeds 100 participants [16–18]. In confirmatory factor analysis (CFA), a rule of thumb is to consider the ratio between cases and free parameters. A range of 10:1 to 20:1 is often suggested [19–21]. Based on these recommendations, the CAST instrument (version 1/ PHASE I) was first tested on 150 participants, then the modified CAST instrument (version 2/ PHASE II) was tested on a second sample of 220 participants.

These participants were all outpatients seeking treatment for a mental or addictive disorder. The primary inclusion criterion for participation in the study was recent cannabis use within the last 12 months. No specific exclusion criteria were applied, except for individuals who were below 18 years of age.

### Measures

The CAST consists of six items, and it utilizes a 4-point Likert scale. The response options range from 0 to 4, with 0 representing “never,” 1 representing “rarely,” 2 representing “occasionally,” 3 representing “quite often,” and 4 representing “very often.” Alternatively, a binary response modality is available, where participants can respond with 1 for “Yes” or 0 for “No” [12].

The CAST is specifically designed for patients who have used cannabis within the last 12 months and is administered in both general practice and in distinct age categories ranging from 15 to 45 years [22].

The study was conducted in two phases. During phase I, the original version (version 1) of the CAST was tested on 150 cannabis-using patients between February 2020 and April 2021. In phase II, the same version of the CAST already distributed in phase I and containing the same number of items after the AFE, was distributed to 220 cannabis-using patients between May 2021 and June 2022.

### Translation and adaptation

The CAST scale has been the subject of a reformulation process, beginning with its translation from French to Moroccan Arabic dialect. Following the initial translation, a panel of experts, including authors of this manuscript (psychiatrists, psychologists, epidemiologists, nurses, and PhD students) revised the scale. To ensure accuracy, the revised version was then back translated into French by two independent translators who were not familiar with the CAST scale. French experts critically reviewed the back-translation and provided valuable feedback, which led to necessary corrections and improvements in the translated version. Once deemed satisfactory, the committee reached a decision on the final Moroccan Arabic dialect version of the scale.

Subsequently, the scale underwent a pilot test involving 10 persons with cannabis use. During this test, the participants completed the questionnaire and provided feedback. It was determined that the scale was comprehensible and not confusing, as no difficulties were reported. No further revisions were made after the pilot test.

### Statistical analyses

All statistical analyses were carried out using the R program. Specifically, the following packages were utilized: “psych”, “corrplot”, “lavaan”, “lavaanPlot and “semTools”, “. Descriptive statistics were used to summarize the characteristics of the participants. To ensure the factorization of the correlation matrix, the suitability of the matrix was assessed using two statistical tests: the Kaiser-Meyer-Olkin (KMO) test and Bartlett’s sphericity test [23]. EFA was employed to investigate the factorial structure of the CAST on the initial sample (n_1_ = 150). Principal axis factoring (PAF) with Promax rotation was used as the extraction method. Items with low communalities (less than 0.40), significant cross-loadings, or loading patterns that did not adequately represent the underlying construct were systematically removed in a stepwise manner. Cronbach’s alpha (CA) coefficient was used as a measure of reliability. The theoretical model of the CAST scale was tested using CFA.

The CFA was conducted on the sub-sample from phase II of the study (n_2_ = 220), encompassing all six items of the scale. To evaluate internal consistency, composite reliability (CR) was calculated. Convergent validity was assessed by calculating the average variance extracted (AVE) as a measure of construct representation, and by comparing CAST scores with those obtained from a gold standard, the Moroccan Arabic version of the Mini International Neuropsychiatric Interview (MINI), in particular the substance-related disorders (non-alcoholic) section [24]. given the absence of a specific measure for cannabis use disorders in our context. For discriminant validity it was examined using two metrics: the Fornell-Larcker criterion [25] and the heterotrait-monotrait ratio (HTMT) [26, 27].

Regarding criterion validity, we used Receiver Operating Characteristic (ROC) analysis to find the optimal threshold for CAST compared to the MINI scale. Sensitivity and specificity were key indicators for identifying optimal scores, and the Youden Y index (Y = sensitivity + specificity − 1) helped balance these measures. The Area Under the Curve (AUC) was decisive in assessing CAST’s ability to distinguish between people with and without a diagnosis, with a higher AUC (tending towards 1) indicating better discriminatory power [28].

### Ethical aspects

All participants were required to give their informed consent before taking part in the study. The study protocol, including the research methodology and ethical considerations, was submitted to the ethics committee of the Hassan II University Hospital of Fez for review and approval (Reference Number: 17/21 – September 2021). All the procedures were followed in accordance with the relevant guidelines and regulations (e.g., Declaration of Helsinki).

To conduct the study at the Fez addictology center for both Phase I and Phase II, two authorizations were obtained from the regional health and social protection directorate. These authorizations were necessary to ensure the study’s adherence to local regulations and guidelines.

## Results

### Sociodemographic characteristics

Our study population, drawn from an addictology center in Fez city, was divided into two samples. A total of 400 questionnaires were distributed, with 160 distributed for phase I and 240 for phase II. After filtering the data and removing invalid or unreliable observations, 150 valid responses were retained for phase I and 220 for phase II.

The two samples in our study exhibited similar sociodemographic characteristics. The mean age was 27.66 ± 7.96 (range 15–45) and 26.99 ± 7.94 (range 15–46), respectively, for the first and second samples. The dominant gender is male, representing 89% in the first sample and 85.90% in the second. Regarding marital status, 74% of participants in the first sample were unmarried, compared to 74.54% in the second sample. In both samples, approximately two-thirds of the participants had completed secondary education, accounting for 60.67%, and 57.27%, respectively. The majority of respondents were from urban areas: 84.67% in the first sample, and 79.10% in the second (Table 1*).*

**Table 1:** Characteristics of participants (*N* = 370)

	Phase I (n_1_ = 150)			Phase II (n_2_ = 220)	
	Mean	n (%)		Mean	n (%)
Age*	27.66 ± 7.96			26.99 ± 7.94	
	(Range 15–45)			(Range 15–45)	
***Sexe***					
Homme		133 (89.00)			189 (85.90)
Femme		17 (11.00)			31 (14.10)
***Marital status***					
Unmarried		111 (74.00)			164 (74.54)
Married		31 (20,67)			42 (19.10)
Divorced		8 (5.33)			14 (6.36)
***Education***					
Illiterate		3 (2.00)			4 (1.82)
Primary education		28 (18.67)			38 (17.27)
Secondary education		91 (60.67)			126 (57.27)
Higher Education		28 (18.66)			52 (23.64)
***Living environment***					
Rural		8 (5.33)			17 (7.72)
Urban		127 (84.67)			174 (79.10)
suburban (village)		15 (10.00)			29 (13.18)

*(Mean ± SD)

### Exploratory factor analysis results

Before conducting the EFA, sampling adequacy and factorability were estimated by the Kaiser-Meyer-Olkin test and Bartlett’s test for the first sample. The total KMO value was 0.79, and all KMO values for each individual element ranged from 0.72 to 0.85, well above the permissible limit of 0.60 [29, 30]. Bartlett’s test of sphericity (χ2 = 282.313, df = 15, *p* < 0.001) demonstrated that the interitem correlations within the data were substantial enough to proceed with the EFA.

The scale’s factor number was established through examination of the scree plot, Horn’s parallel analysis, and adherence to the eigenvalue greater than one criterion [31]. EFA was performed using PAF extraction method with “promax” rotation to determine the factor structure of the scale. The PAF, one of the most effective estimation methods in EFA, was selected for several advantages. First, PAF does not rely on distributional assumptions [32]. Second, PAF demonstrates greater robustness in situations with unequal factor loadings, limited indicators per factor, and small sample sizes [33]. Finally, PAF excels in recovering weak factors, a quality shared a few other methods [33, 34].

A saturation threshold of at least 0.40 was applied. Items whose loadings did not surpass this threshold or loaded significantly on multiple factors were excluded from factors. After each iteration, the rotated factor matrix analysis displayed significant loadings and alterations in communality values.

As a result, a model consisting of three factors of the Moroccan version of the CAST subscales was adopted rather than sticking to a single-factor model, which was analyzed in depth. The unsatisfactory results of the single-factor model, as shown by fit indicators (e.g., CFI = 0.78; 216 TLI = 0.64; NFI = 0.77; GFI = 0.94; RMSEA = 0.26; SRMR = 0.07) reinforced the choice of the three-factor model.

The first factor, explaining 22% of the variance, comprises two items (smoking alone and smoking before noon) named “use patterns [UP]”, which pertain to the initiation phase of cannabis consumption, whether solitary or in a social context. The second factor, termed “use reduction [UR]”, explains 22% of the variance and is represented by two items (friends or family and attempted to reduce or stop) that address attempts to reduce cannabis consumption. Last, the third factor, “use disorders [UD]”, accounts for 26% of the variance and is loaded by two items (memory disorders and problems) related to potential disorders that may emerge because of cannabis use.

These three factors were then evaluated using CFA. With eigenvalues of 0.202, 0.130 and 3.502 for “UP”, “UR” and “UD” respectively, the factors each comprised two elements and represented a total variance of 70% (Table 2).

**Table 2:** Factor structure of the Moroccan version of CAST (6 items)

Items	Factors			h^2^	Item-total correlation	Alpha
	UP	UR	UD			
Smoking alone (UP1)	**0.72**	0.08	0.02	0.62	0.77	0.83
Smoking before noon (UP2)	**0.77**	0.00	0.01	0.61	0.75	0.84
Friends or family (UR1)	-0.06	**0.80**	0.06	0.78	0.76	0.84
Attempted to reduce or stop (UR2)	0.21	**0.73**	-0.05	0.63	0.74	0.84
Memory disorders (UD1)	-0.11	0.13	**0.88**	0.73	0.79	0.83
Problems (UD2)	0.18	-0.11	**0.85**	0.82	0.78	0.83
*Eigenvalue*	0.64	0.33	3.23			
*Variance (total = 70%)*	22%	22%	26%			

#### Test of reliability

The construct’s internal consistency and reliability were assessed using Cronbach’s alpha coefficient. Cronbach’s α and item-total correlations were calculated for each construct and individual item, as shown in Table 2. The reliability statistics provide the true value of the overall Cronbach’s coefficient (α = 0.86). Additionally, the alpha values for the items within each subscale ranged from 0.83 to 0.84, indicating a high level of internal consistency. These results confirm that the Moroccan version of the CAST in our sample demonstrated strong internal consistency. It is noteworthy that alpha values of at least 0.70 and ideally above 0.80 are considered indicative of good consistency [35, 36]. Therefore, the obtained alpha values suggest that all the concepts assessed in the study were reliable.

### Confirmatory factor analysis

#### Interscale correlations

The three factors exhibited strong and statistically significant correlations (*p* < 0.001). The highest correlation was observed between “UP” factor and the “UR” factor (*r* = 0.77). Furthermore, the “UD” factor showed a positive correlation with the “UP” factor (*r* = 0.66) and was also positively correlated with the “UR” factor (*r* = 0.56) (Table 3).

**Table 3:** Results of composite reliability, average variance extracted, and correlations between latent constructs

	CR	AVE	Latent constructs		
			UP	UD	UR
Use Patterns (UP)	0.76	0.62	**0.79**		
Use Disorders (UD)	0.88	0.78	0.66^a^	**0.88**	
Use Reduction (UR)	0.81	0.67	0.77^a^	0.56^a^	**0.82**

a *p* < 0.001

**CR:** Composite reliability, **AVE:** the square root of the average variance extracted

#### Convergent validity

**Fig. 1:**
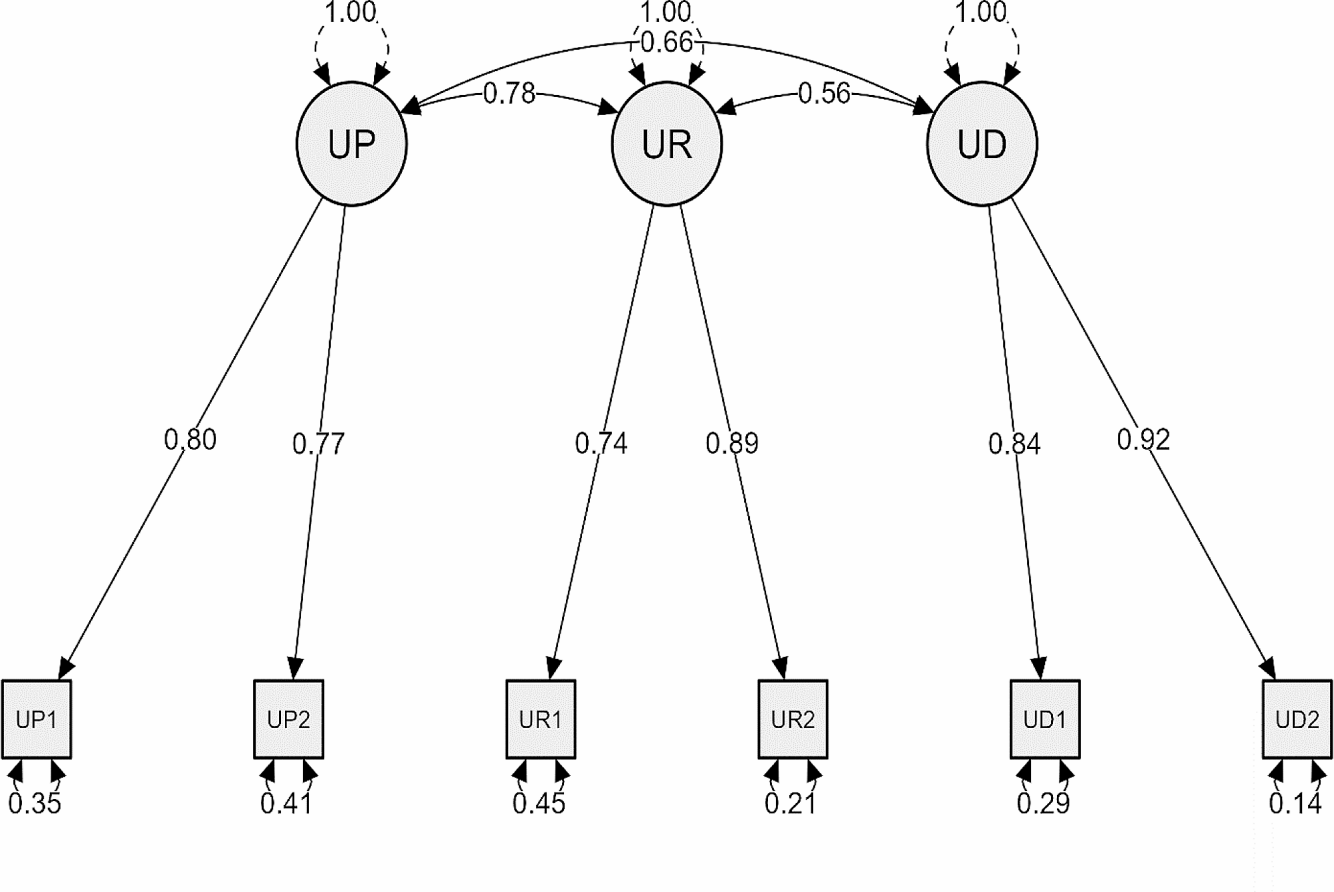
CFA measurement model

The findings from the CFA also demonstrated that the standardized regression coefficients were above 0.70, and the factor loadings for the “reduced consumption” factor (UR1) were the lowest, with a value of 0.74. Conversely, the loadings for the other six factors were all greater than 0.77. Additionally, the t-ratios, computed by dividing the parameter estimate by the standard error, were greater than 1.64 for each factor-factor and factor-variable pair. These t-ratios indicated significant relationships between the variables, with *p* values below 0.001, signifying a high level of statistical significance. Therefore, considering the regression coefficients exceeding 0.50 and the significant relationships associated with the high t scores, the first-order CFA offered statistically acceptable evidence of convergent validity [23] (Fig. 1*).*

Moreover, to confirm the reliability and convergent validity of the instrument, the composite reliability (CR) and average variance extracted (AVE) indicators were calculated [37]. With CR values between 0.76 and 0.88 and AVE values ranging from 0.62 to 0.78, the results of the entire factor analysis process were confirmed, suggesting a favorable fit of the CAST instrument to the collected data (Table 3*).*

Similarly, the correlation analysis between the detection outcomes of the validated instrument (CAST) and the Gold Standard instrument (MINI) indicated a statistically significant and relatively high correlation (*r* = 0.81, *p* < 0.001), consistent with the CR and AVE (Table 4*).*

**Table 4:** Correlation of CAST score and MINI score

	MINI			
	Pearson’s r	*p*	Lower 95% CI	Upper 95% CI
***CAST***	0.81***	< 0.001	0.76	0.85

* *p* < 0.05, ** *p* < 0.01, *** *p* < 0.001

#### Discriminant validity

To assess the discriminant validity of the model, which consists of measuring the degree of differentiation between overlapping concepts [38], two criteria were used: the Fornell & Lacker criterion and the HTMT [26, 27, 37].

Table 3 presents the intercorrelations between the dimensions of the latent factors, with the square root of the average variance extracted (AVE) values highlighted in bold. Among the correlations, the highest value (0.75) was observed between the factor’s “UR” and “UD”, while the lowest value among the square roots of the AVE values was 0.82. Notably, the diagonal values of the matrix were greater than the off-diagonal values in the corresponding rows and columns [25].

The HTMT criterion value should be below 0.85 for strict [39, 40] and 0.90 for liberal discriminant validity [41, 42]. Table 5 reveals that all matrix values are below 0.85, providing further support for the potential discriminant validity among all the concepts in the proposed model. Overall, the reliability tests and tests for convergent and discriminant validity consistently support the justification of the concepts in the measurement model based on both types of tests (Fornell and Larcker criterion and HTMT).

**Table 5:** Discriminant validity (HTMT Criterion)

	Latent constructs		
	UP	UD	UR
Use patterns (UP)	**1**		
Use disorders (UD)	0.65	**1**	
Use reduction (UR)	0.77	0.60	**1**

#### Fitness of the measurement mode

The results of the CFA showed that the fit indices for the three-factor model were good (Table 6*).* Specifically, the chi-square to degrees of freedom ratio (χ2/df) was 2.23, indicating an acceptable fit [43, 44]. The comparative fit index (CFI) = 0.99 (> 0.90) suggests that the fitted model is in very good agreement with the observed data [45]. The goodness-of-fit index (GFI) was 0.99 (> 0.90), reflecting a high level of model fit [46, 47]. The standardized root mean square residual (SRMR) was 0.02 (< 0.05), suggesting a small discrepancy between the model and the observed data [48, 49]. The standardized root mean square residual (RMSEA) was 0.07 (< 0.08), suggesting a reasonable fit between the model and the data [50–52]. The normed fit index (NFI) was 0.98 (> 0,90), and the non-normed fit index (NNFI) or Tucker-Lewis Index (TLI) was 0.97 (> 0,90) [46, 47].

**Table 6:** Fit indices

Fit index	χ^2^	χ^2^/df	CFI	GFI	RMSEA	SRMR	NFI	TLI
*Observed Value*	*13.40* *p* = 0.037	2.23	0.99	0.99	0.07	0.02	0.98	0.97
*Level of acceptance*	*p* > 0.05	< 3	> 0.90	> 0.90	< 0.08	< 0.05	> 0.90	> 0.90

**χ**^**2**^**:** Chi-squared test; **df:** Degrees of Freedom; **CFI:** Comparative fit index; **GFI:** goodness of fit index; **RMSEA:** root mean square error of approximation; **SRMR:** Standardized Root Mean Square Residual; **NFI:** normed fit index; **TLI:** Tucker-Lewis Index

#### Detection capability

The examination of CAST’s detection capacity using the MINI as the Gold Standard reveals high sensitivity and Positive Predictive Value (PPV) for CAST, both exceeding 0.90. However, the specificity and Negative Predictive Value (NPV) are relatively low. The optimal balance between sensitivity and specificity for the CAST scale is achieved at a cutoff of 3 and 4, identified by the maximum Youden index (Y = 0.75 and Y = 0.73) (Table 7*).*

**Table 7:** Screening characteristics of CAST across different cutoff points

CAST					
Cut-off	Se (%)	Sp (%)	PPV (%)	NPV (%)	Y
1	100	0	96.4	-	0.00
2	100	25	97.2	100	0.25
3	99.53	75	99.06	85.71	0.75
4	97.64	75	99.04	54.55	0.73
5	93.87	75	99	31.58	0.69
6	91.04	75	98.97	24	0.66

**Se: **sensitivity, **Sp:** specificity, **PPV:** positive predictive value, **NPV:** negative predictive value, **FP:** false positives, **FN:** false negatives, and **Y:** Youden index

**Fig. 2:**
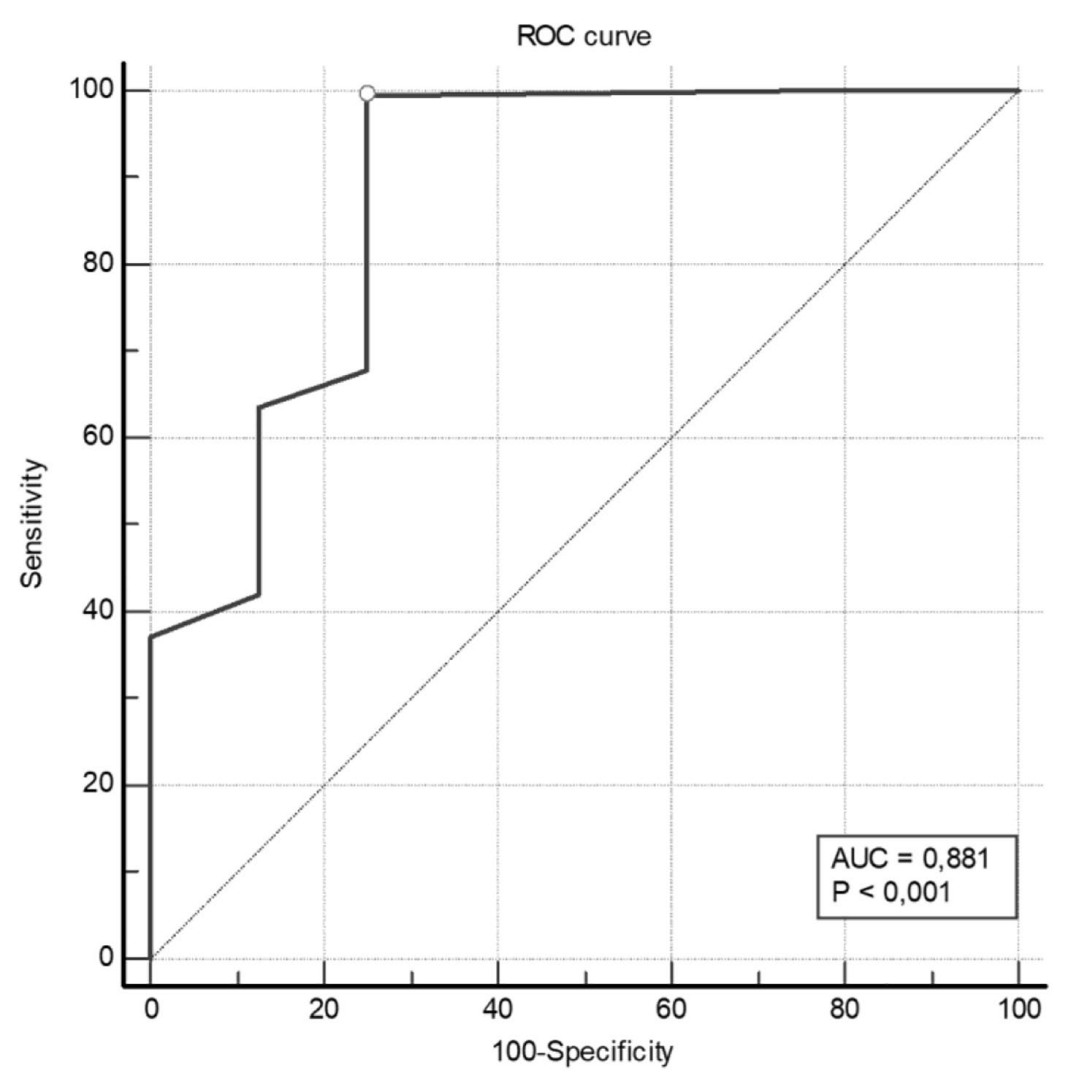
ROC curve and AUC for CAST

The high discriminatory power of the scale is evident in the high AUC of 0.881 (95% IC: 0.83–0.92) revealed in the CURVE ROC (Fig. 2), which affirms its strong ability to distinguish individuals with a clinical diagnosis from those without it.

## Discussion

The purpose of this study was to assess the psychometric properties of a Moroccan version of the CAST, a scale designed to identify patterns of cannabis use that may lead to adverse health or social consequences for individuals who use cannabis. Two samples comprising 150 and 220 persons with cannabis use were examined. The participants were recruited from an addictology center in the city of Fez. The average age of the participants was 26.99 ± 7.94 (range 15–46) (range 15–46), with a notably early age of center follow-up at 15 years. The sample predominantly consisted of males, making up 85.90% of the participants, while females accounted for 14.10%. These sociodemographic characteristics align with the prevailing Moroccan and global trends, wherein there is a rising prevalence of substance use among adolescents aged 13 to 17 [4, 6], and higher rates of drug use or dependence among men across various age groups [53, 54]. The participants in our sample share similarities in demographic characteristics, such as age and sex, with those observed in studies conducted outside of Morocco. However, there are significant differences between our sample and the samples used to validate the CAST in terms of economic and sociocultural dimensions.

The Moroccan adaptation of CAST validated its internal structure using a robust three-factor model that revealed specific aspects of cannabis use among the individuals assessed. The first factor, called ‘consumption habits’, relating to ‘consumption patterns’, could prove crucial in understanding non-recreational consumption habits, encompassing more compulsive or solitary behaviours and habits such as ‘smoking alone’ or ‘smoking before noon’. The second factor, relating to ‘use disorders’, could play a key role in identifying the negative implications for memory and general problems that may arise from cannabis use (‘memory disorders’ or ‘general problems’). Finally, the third factor, concerning ‘reduction of consumption’, offers an insight into the efforts made by individuals ‘attempting to reduce or stop’ or those around them ‘close friends or family’ to change their consumption habits in order to eventually attempt to reduce consumption. The significant correlation observed between these three factors underlines the resilience and coherence of the scale’s internal structure.

This structure of the Moroccan version of the CAST differed from the structures found in previous studies, with studies in Spain [13, 22, 55, 56] revealing a bidimensional structure and other studies of French adolescents [57] or Hungarian pupils and students [58] identifying a unidimensional structure. However, the first factor identified in our “use patterns” structure is consistent with the first factor identified in other studies with a two-dimensional structure with the same items (“smoking alone”, “smoking before noon”) [13, 22, 55, 56]. Unlike the other two factors identified, “use disorders” and “use reduction” were replaced by a single factor in the same studies with a bidimensional structure.

The internal consistency indicators obtained for the CAST were high and surpassed those obtained in certain previous studies [13, 57, 59, 60]. The confirmatory factor analysis (CFA), in our study, showed that the goodness-of-fit indicators for the three-factor model are similar to those of the two-factor model in the Spanish and French version structure [13, 61], and slightly better than those of the one-factor models [13].

Our results suggest that the optimal cut-off scores for identifying problematic cannabis use in the young adult population are 3 and 4 points for the CAST. These findings are consistent with the results reported by Legleye [12, 60] and Rial [13]. At these cut-off points, both scales exhibit high sensitivity values and generally lower but still elevated levels of specificity. The selection of these empirical cut-off scores is grounded in the Youden Index, ensuring the best balance between sensitivity and specificity. The relevance of using the suggested criteria varies according to the specific objectives of the research using CAST [60]. However, when we plan to include young participants, it is preferable to opt for a CAST score of 3 points because of its greater sensitivity at this threshold [13].

Although some existing measures use a one factor [12, 13, 60] or two-factor structure [13, 22, 55, 56] for an overall assessment of cannabis use, the choice of a three-dimensional approach in the Moroccan CAST takes into account the complexity and diversity of cannabis-related behaviours. Research has shown that the three-dimensional model provides a more nuanced understanding of substance use patterns. The three factors identified - ‘patterns of use’, ‘use disorders’ and ‘use reduction’ - allow a comprehensive exploration of the different aspects of cannabis use that might be overlooked in a single-factor model. Moreover, adopting a tridimensional approach, as seen in the Moroccan CAST, offers practical implications for tailored interventions and prevention strategies.

While the measurement tool exhibited commendable internal consistency and satisfactory adequacy indicators across its three factors, its validity confronts notable limitations influenced by contextual elements, population specificity, timing, and score utilization, particularly in studies characterized by small sample sizes, as evident in our case. Additionally, despite the existence of translated and valid scales in our context to assess drug addiction as a whole [24, 63], the absence of valid and specific measurements in Moroccan Arabic to assess problems of cannabis consumption prevented our study from explicitly examining incremental validity. This could be acknowledged as a constraint in understanding the scale’s potential contribution beyond existing measures. Attempting to generalize findings from research conducted in a specific locale with a unique population may encounter hurdles when extrapolated to another sample from a different location or demographic group. Therefore, exercising caution in interpreting and applying the results in diverse contexts is essential, recognizing that the effectiveness of the measurement tool may fluctuate depending on the characteristics of the studied population and the specific context.

## Conclusion

In summary, the present study provides strong evidence supporting the psychometric properties of the Moroccan version of CAST as an effective tool for identifying problematic cannabis use. Although some limitations exist, the CAST remains a valid and reliable instrument that is well suited for early cannabis use screening. Its significance extends to prevention policies, making it suitable for both mass periodic screening and individual case identification.

## Data Availability

The datasets used and analyzed during the current study are available from the corresponding author on reasonable request.

## Abbreviations

χ2/ df: Chi square value/degrees of freedom
AGFI: Adjusted goodness-of-fit index
AUC: Area Under the Curve
AVE: Square root of the average variance extracted
CA: Cronbach’s Alpha
CAST: Cannabis abuse screening test
CFA: Confirmatory factor analysis
CFI: Comparative fit index
CR: Composite reliability
EFA: Exploratory factor analysis
FA: Fatalism
GFI: Goodness of fit index
HTMT: Heterotrait-Monotrait ratio
KMO: Kaiser-Meyer-Olkin
MeDSPAD: Mediterranean school survey project on alcohol and other drugs
MINI: Mini International Neuropsychiatric Interview
NFI: Normed fit index
NNFI: Non-normed fit index
NPV: Negative Predictive Value
PAF: Principal axis factoring
PPV: Positive Predictive Value
RMSEA: Root mean square error of approximation
ROC: Receiver Operating Characteristic
SRMR: Standardized Root Mean Square Residual
TLI: Tucker-Lewis Index
UD: Use Disorders
UNODC: United Nations Office on Drugs and Crime
UP: Use Paterns
UR: Use reduction
